# Effects of Medical Education Program Using Virtual Reality: A Systematic Review and Meta-Analysis

**DOI:** 10.3390/ijerph20053895

**Published:** 2023-02-22

**Authors:** Hyeon-Young Kim, Eun-Young Kim

**Affiliations:** 1 College of Nursing, Sahmyook University, Seoul 01795, Republic of Korea; 2 VR Healthcare Content Lab, Seoul 01795, Republic of Korea; 3 Asan Medical Center, Seoul 05505, Republic of Korea

**Keywords:** virtual reality, health personnel, unlicensed, randomized controlled trial, systematic review

## Abstract

Several studies have examined the effect of virtual reality (VR) education. However, they are mostly systematic reviews or meta-analyses focusing on doctors and residents; they fail to consider VR medical education for a broader range of learners. We evaluated the effectiveness of VR education for health professionals and identified the essential features of education. Randomized controlled trials published from January 2000 to April 2020 were identified from PubMed, Embase, CINAHL, and the Cochrane Library (n = 299). The randomized studies’ bias risk was evaluated using Cochrane’s Risk of Bias tool. Meta- and subgroup-analyses were conducted using Review Manager 5.4.1. The overall effect was measured using Hedges’ g and determined using Z-statistics (*p* < 0.05). Heterogeneity was assessed using X^2^ and I^2^ statistics. Among the identified records, 25 studies were selected through systematic review, and 18 studies were included in the meta-analysis. We identified a significant improvement in the VR group’s skill and satisfaction levels, and that less immersive VR was more efficacious for knowledge outcomes than fully immersive VR. Maximizing the advantages of VR will increase learning opportunities and complement the limited clinical experience, thus improving medical services. A systematic and efficient VR medical education program will greatly enhance learners’ core competencies.

## 1. Introduction

Virtual reality (VR) has a positive impact on learning as it allows the user to become completely immersed in an interactive virtual environment similar to the real environment. VR creates a user environment with a 3-D virtual background using advanced computer graphics and various displays and interfaces [1]. VR is therefore widely applied in many areas and industries [2], including education, where it is positioned as a tool to deliver individualized, cooperative, and problem-solving learning experiences [3]. 

High-fidelity simulators have been adopted in the field of education, but there are considerable financial, supervisory, and spatial restrictions involved in using them [4,5]. In contrast, VR is emerging as an alternative educational tool as it is more economical and needs less space compared to traditional educational settings [6,7], prioritizes safety without causing harm to the subjects and learners, and allows learners to learn from experience in an environment similar to a clinical environment [6,7]. However, as there is significant cost and effort involved in designing VR programs in the early stage, as well as in the training of educators, research is needed to provide reliable evidence of its effectiveness by comparing it with existing education methods and analyzing the costs involved in the active introduction of VR programs.

VR programs place learners in a key role through the exercise of their complex cognitive thinking skills, psychomotor control, performance, and communication skills [8]. There is a necessity for a systematic review or meta-analyses that examines the effectiveness of VR programs as a learning strategy in the acquisition of clinical performance in medical education and how this innovative learning strategy affects skill, knowledge, self-efficacy, satisfaction, and anxiety in health personnel.

Numerous studies confirming the effects of VR education have been conducted, but they are mostly systematic reviews [9,10] or meta-analyses related to doctors and residents. They exclude prospective health personnel and generally fail to provide evidence of the application of VR medical education for a broad range of learners [11,12,13].

Therefore, this study reviews the literature on the conduct of medical education programs using VR for current and prospective health personnel. We specifically evaluate their effectiveness to ascertain the optimal method for educating health personnel, identify the necessary features of medical education, and provide direction for future research pertaining to the development of VR-based medical education programs.

## 2. Materials and Methods

### 2.1. Study Design

This study is a systematic review and meta-analysis that was conducted to integrate and determine the effects of VR-based medical education on current and prospective health personnel. This study was conducted according to the reporting guidelines outlined in the Preferred Reporting for Systematic Reviews and Meta-Analysis (PRISMA) statement [14].

### 2.2. Search Strategy

The data search was conducted using the following databases: Pubmed, Embase, CINAHL, and the Cochrane Library. The search was conducted between March 2020 and April 2020 and was limited to data that was published from January 2000 to April 2020. Two independent reviewers with previous experience in handling search-extracted data for meta-analyses screened the articles using the title/abstract and full text. They removed duplicates and screened the reference lists of eligible articles and relevant systematic reviews. 

The search formula was constructed using 8 related Medical Subject Headings (MeSH) terms after a database search according to the key question strategy, PICOSD (Participants, Intervention, Comparison, Outcomes, Study Design). The identified keywords and MeSH were combined using the following combinations of terms with the Boolean operator “OR”: “Students, Nursing”; “Students, Medical”; “Students, Dental”; “Nurses”; “Physicians”; “Internship and Residency”; “Virtual Reality”; and “Augmented Reality”. “Participants” and “Intervention” were ascertained using the Boolean operator “AND”. Further details on the search strategy are provided in Supplementary Materials. For a comprehensive data search, only studies published in English were considered.

### 2.3. Inclusion Criteria

The sample included participants (P) who are current and prospective health personnel and intervention (I) concerned with an educational intervention using VR. Comparison (C) pertained to papers with no training, conventional patient-based training, standard education, and traditional education. Outcomes (O) targeted papers that measured the skills, knowledge, self-efficacy, satisfaction, and anxiety of the participants using a measurement tool that evaluated the effect of education. Study design (SD) included a Randomized Controlled Trial (RCT), or cluster RCTs (cRCTs), each being an intervention study including a control group. 

### 2.4. Study Selection

From the searched literature, duplicate data were removed with the help of RefWorks, a web-based bibliography and database manager. The title and abstract of the related articles were first checked, along with the main text, and the articles were selected based on the selection criteria and were reviewed. During this process, some papers were excluded and the reasons for exclusion were specified. A total of 25 papers were finally selected to extract the general characteristics, interventions, and research results. After data extraction, it was cross-checked, and, if there was no agreement, the original text was reviewed together to increase the accuracy of the data. 

### 2.5. Data Extraction

Data were extracted according to the standard methods of data extraction specified in the Cochrane Handbook for Systematic Reviews of Interventions [15]. From the selected studies, we extracted the following information: the year of publication, country, study design, number of participants, demographic characteristics of study participants, type of education provided, number, total time of intervention, the type of equipment used to implement VR, the degree of immersion according to the type of equipment (including VR glasses, other types of Head Mounted Displays [HMD], Cave Automatic Virtual Environments [CAVE], and Oculus Rift), the educational method applied to the control group, outcome variables, and tools.

### 2.6. Risk of Bias

In the randomized control group, the quality was rated as low (+), high (−), and uncertain (?). The risk of bias was ascertained using Cochrane’s Risk of Bias (RoB) tool that included six domains. As part of the quality assessment, the reasons for the judgment were described in accordance with the assessment framework of the RevMan program.

### 2.7. Synthesis of Results

The general characteristics of research papers were presented in terms of frequency and average. For studies in which meta-analysis was possible, the effect size and the homogeneity of educational interventions were determined using VR applied to current and prospective health personnel. They were calculated using Review Manager 5.4.1 (Cochrane Library Software, Oxford, UK). For outcome variables with different effect sizes, standardized mean difference (SMD) was chosen as the method of analysis. Furthermore, research articles that provided mean values or standard deviations and test statistic values or confidence intervals were included to calculate the effect size of the experimental and control groups. A fixed effects model was used to merge the effect sizes with confirmed homogeneity under the assumption that the outcome variables of each study were the same. Confirmed heterogeneity was calculated using a random effects model, and homogeneity was confirmed using Cochrane’s chi-square test and the I^2^ test: 0~25% indicated low heterogeneity, 25~75% indicated moderate heterogeneity, and 75~100% indicated significant heterogeneity [16]. For the subgroups that showed high or moderate heterogeneity, a moderating effect analysis (i.e., a subgroup analysis to identify differences in effect size by group) was performed to identify the cause of the variance, focusing on the variance in the effect size. The statistical significance of the effect size (d) was determined using the overall effect test and the 95% confidence interval (CI), with a 5% significance level. Effect sizes were interpreted based on SMD [17]: SMDs of 0.20 are “small” in magnitude, those around 0.50 are “medium,” and those around or above 0.80 are considered “large”. To determine publication bias, statistical analysis using Egger’s regression test was performed in the case of 10 or more studies included in the analysis along with a funnel plot. 

## 3. Results

### 3.1. Characteristics of the Research Targets (Study Selection)

**Figure 1:**
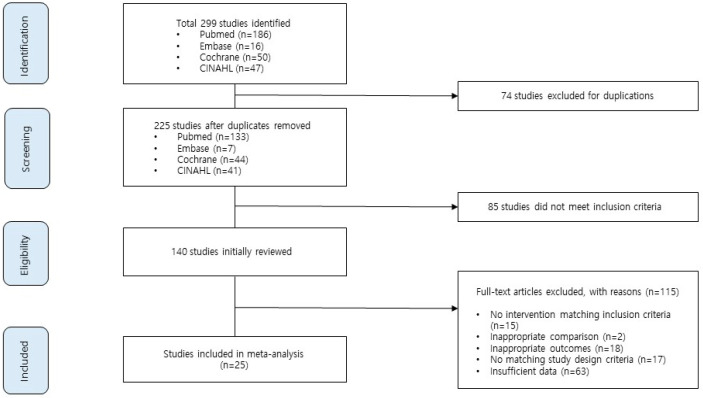
Flow chart of study selection for the analysis of the effects of medical education program using virtual reality.

The results of the search are shown in Figure 1. A total of 299 papers were retrieved by searching the PubMed, Embase, CINAHL, and Cochrane Library databases; titles and abstracts were reviewed with inclusion and exclusion criteria in mind, and 140 articles were selected. After excluding 115 papers that did not meet the inclusion criteria, 25 papers were finally selected.

### 3.2. Characteristics of Educational Intervention Studies Using VR Conducted on Current and Prospective Health Personnel (Study Characteristics)

The general characteristics of 25 educational intervention studies using VR were included in this study; the details are presented in Table 1. As far as the characteristics of the study design are concerned, all 25 studies were randomized control studies. The number of study participants was 546 in the experimental group and 506 in the control group–1052 in total. Fifteen studies were conducted on prospective health personnel, eight studies were on health personnel, and two studies included both current and prospective health personnel. Of the total 25 studies, 18 were conducted on doctors or medical students, two were conducted on prospective dentists, and five on nursing students.

### 3.3. Details of Training

The details and VR equipment used in the 25 VR interventions are illustrated in Table 1. According to the degree of immersion, the studies can be divided into 20 less immersive VR and 5 fully immersive VR interventions. According to the type of educational intervention, 17 were skills-oriented, seven were scenario-based, and one was theory-oriented.

More specifically, 17 skills-oriented interventions included five laparoscopies, two fiberoptic bronchoscopies, two arthroscopy or arthroscopic knee surgery, one colonoscopy, one ureteroscopy, four surgical skill for doctors or medical students, one cavity training for dental students, and one intravenous catheterization for nursing students. The seven scenario-based interventions included one triage training and one endoscopic performance for doctors or medical students, one dentistry simulation for dental students, one disaster training, one virtual patient simulation for pediatrics, one clinical virtual simulation case-based learning, and one operating room fire drill scenario for nursing students. There was one theory-oriented intervention and one intervention on neuroanatomy for doctors and medical students. 

The number of interventions varied from 1 to 25 times (average = 4.13) and the duration of interventions ranged from 10 min to 240 min (average = 81.76).

### 3.4. Methodological Quality Assessment of Intervention Studies

**Figure 2:**
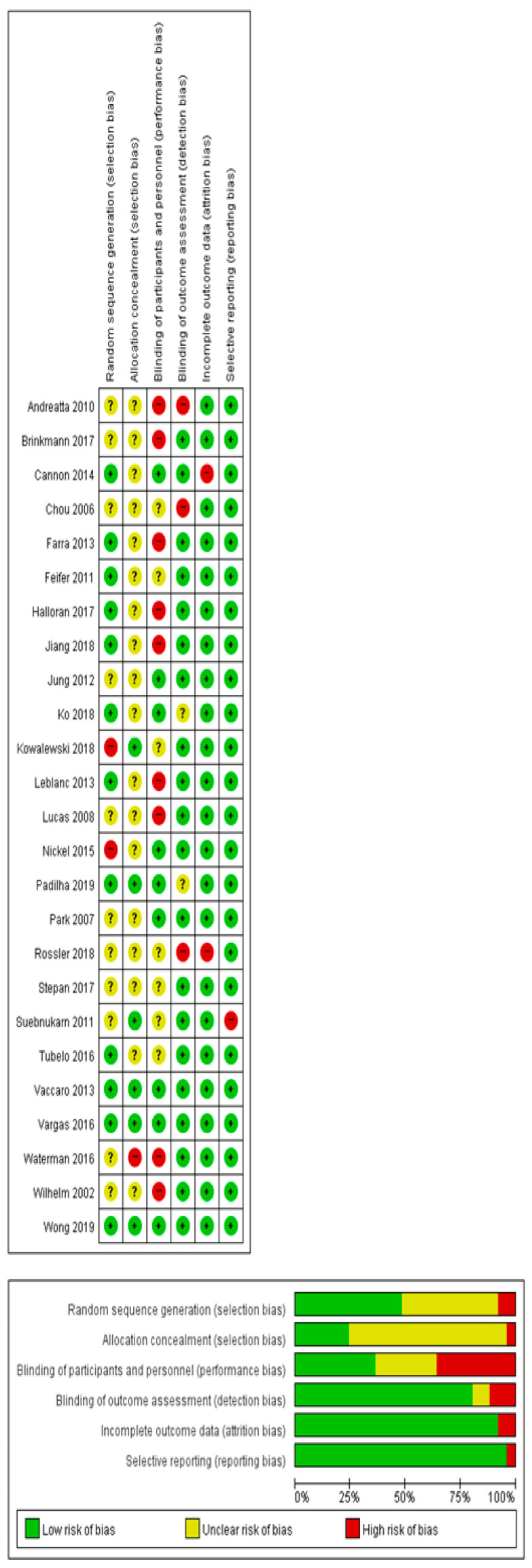
The risk of bias graph for included studies.

The risk of bias assessment is summarized in Figure 2. Details pertaining to randomization were described in 12 out of 25 studies, and a lack of clarity on assignment concealment was found in most of the studies (18 out of 25). The blinding of participants and researchers was described in detail in nine studies, and the blinding of raters was described in 20 studies. In 23 studies, the incomplete outcome data was described in detail, and in 24 studies, selective outcome reporting was described (Figure 2). 

### 3.5. Effect of Educational Intervention Using VR

#### 3.5.1. Effect Size According to Skills

Eighteen RCT studies evaluated skills, and a meta-analysis was conducted on 10 studies; the total number of subjects was 297. A total of 11 studies were analyzed, including the number of applications for different interventions within the same study. As the heterogeneity was shown to be low and moderate (X^2^ = 13.96, *p* = 0.17, I^2^ = 28%), the effect size was calculated using the fixed effect model, and the total effect size of the skills was found to be statistically significant at 0.72 (95% CI: 0.48, 0.96) (*p* < 0.001). As revealed by the funnel plot and Egger’s tests, there was no publication bias in general (*p* = 0.728).

#### 3.5.2. Effect Size According to Knowledge

**Figure 3:**
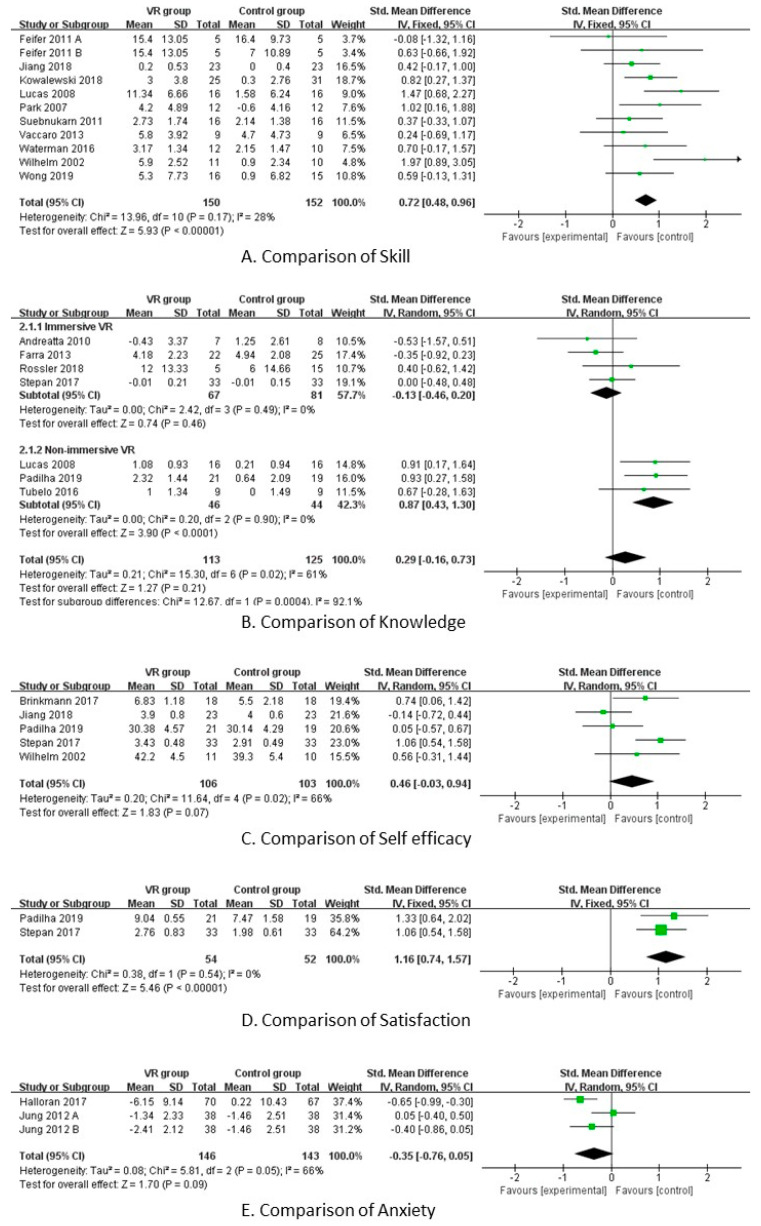
Forest plot of effects of outcomes on virtual reality.

Eight RCT studies evaluated skills, of which meta-analysis could be conducted for seven studies; the total number of subjects was 238. As the degree of heterogeneity was found to be high and moderate (X^2^ = 15.30, *p* = 0.02, I^2^ = 61%), the effect size was calculated using the random effect model, and the total effect size of the knowledge variable was not statistically significant at 0.29 (95% CI: −0.16, 0.73) (*p* = 0.21) (Figure 3). Heterogeneity was explored through subgroup analysis according to the difference in immersion in virtual reality, among the characteristics of the intervention, and the effect size was analyzed to confirm that the four fully immersive groups were homogeneous (X^2^ = 2.42, *p* = 0.49, I^2^ = 0%), and the effect size of knowledge was not statistically significant at –0.13 (95% CI: −0.46, 0.20) (*p* < 0.46). The three less immersive groups were found to be homogeneous (X^2^ = 0.20, *p* = 0.90, I^2^ = 0%), and the effect size of knowledge was statistically significant at 0.87(95% CI: 0.43, 1.30) (*p* < 0.001). The funnel plot did not reveal any publication bias in general.

#### 3.5.3. Self-Efficacy

Five RCT studies evaluated self-efficacy, and only two studies considered pre- and post-test values, so the effect size was analyzed only with the post values; the total number of subjects was 209. As the degree of heterogeneity was found to be high and moderate (X^2^ = 11.64, *p* = 0.02, I^2^ = 66%), the total effect size of self-efficacy was calculated using the random effect model, and the total effect size of knowledge was not statistically significant at 0.46 (95% CI: −0.03, 0.94) (*p* = 0.07) (Figure 3). As revealed by the funnel plot, no general publication bias was found.

#### 3.5.4. Satisfaction

To determine satisfaction level, we analyzed two RCTs from which post-values could be extracted; the total number of subjects was 106. As no heterogeneity was revealed (X^2^ = 0.38, *p* = 0.54, I^2^ = 0%), the total effect size of satisfaction was calculated using the fixed effect model, and the total effect size of satisfaction was found to be statistically significant at 1.16 (95% CI:0.74, 1.57) (*p* < 0.001) (Figure 3).

#### 3.5.5. Anxiety

To examine anxiety, three studies were used for the analysis, which considered the number of applications for different interventions within the same study. The number of subjects was 251. As a high degree of heterogeneity was found (X^2^ = 5.81, *p* = 0.05, I^2^ = 66%), the total effect size of anxiety was calculated using the random effect model, which was not statistically significant at 0.35 (95% CI: −0.76, 0.05) (*p* = 0.09) (Figure 3).

## 4. Discussion

In this study, we conducted a systematic review and meta-analysis of studies that conducted medical education using VR for prospective and current health personnel, to integrate and determine their effects. Of the 25 RCTs, 20 studies were on doctors—these mostly involved skill-based interventions—and only five studies were on nurses, out of which four studies were scenario-based. Future researchers would benefit from analyzing the effects of VR education on medical professionals based on various research results. With respect to the effects of educational intervention, the skill was mostly investigated (10 studies), followed by knowledge (seven studies), self-efficacy (five studies), and satisfaction and anxiety (two each). It was confirmed that skill and knowledge were the most investigated variables to determine the effect of VR interventions. This finding is consistent with that of previous meta-analyses [11,18,19]. All studies analyzed in this paper are well-designed RCTs. Although the analysis involved a small number of studies, this study is significant as it analyzes additional variables that were not covered in previous studies [12], such as self-efficacy, anxiety, and satisfaction. Additionally, the existing meta-analytical study [13] only reported data immediately after the intervention, but this study compared the values before and after the intervention, and hence the evidence can be considered more valid and reliable. 

In this study, the overall effect size for the skill of educational intervention using VR was 0.72, which is a relatively large medium size, and is similar to the results of previous VR meta-analyses where the effect size of the intervention was reported to be 0.90 and 1.12 [13,18]. However, the previous studies suffered from a limitation; that is, while interpreting the meaning of the effect size, there was a high degree of heterogeneity, no control group was included, and only posttest values were analyzed. Hence, the results of this study can be considered significant and more reliable. Medical education programs using VR can be accessed by learners through immersive experiences even in such circumstances in which the safety of the learners and educators cannot be guaranteed due to the spread of infectious diseases or other natural disasters [20,21]. In a VR environment, learners can understand the importance of patient safety and improve clinical skills in a safe environment through repeated learning while modifying the incomplete interventions appropriately [22,23]. As suggested in this study, and as an effective way to improve skills, educational interventions using VR can be considered more realistic and appropriate and should be promoted more aggressively for the practical education of health personnel in an environment where the practice is limited, like in a situation such as the COVID-19 pandemic. Previous studies have reported that the skill score may change with respect to feedback [24,25]. This study found a variety of feedback methods that can be adopted in VR education, but these characteristics were not considered for the analysis, and the main limitation of this study was that the effect sizes were not integrated or interpreted. Future research must be conducted to verify the effectiveness of VR medical education through repeated RCT studies considering the type and characteristics of feedback. This will help in providing the necessary guidelines regarding the type of feedback that can be applied in VR medical education. 

Through the meta-analysis of the knowledge variable, we found that the experimental group had a higher knowledge score than the control group, but with no statistically significant difference, which is in line with the results of previous studies [13,18]. This result may be attributed to the fact that most of the tools that were used for measuring the level of knowledge were not standardized tools developed by researchers. In addition, the period for which the knowledge can be retained also differed with respect to the subjects’ existing knowledge level and the research period. Educational intervention using VR is considered an educational method that facilitates the development of integrated thinking ability and adaptability rather than simply improving knowledge, hence the need to measure critical thinking and integrated thinking ability. As a result of the subgroup analysis of knowledge according to the degree of immersion, the less-immersive VR program was found to have an effect size of 0.87. This finding is in line with that of previous studies that reported that less-immersive VR education was more effective than fully immersive VR education using HMD or VR cave for acquiring knowledge [26,27]. In the case of fully-immersive VR, it is believed that the learning environment and use of complex and cumbersome equipment, to increase the level of immersion, can act as obstacles to the process of cognitive learning. Additionally, learners might experience cybersickness while being fully immersed in the VR environment, and this may act as a barrier to the acquisition of knowledge [28,29]. Therefore, to reduce learners’ cybersickness in a fully-immersive VR, the VR operation time, rest time, and parallel movement method after wearing equipment in designing the program should be carefully considered [30,31]. Since the problem of cybersickness is being overcome with the latest technological advancements and supplements [27], future researchers may conduct repetitive studies in a more stable environment while controlling the intervening variables. As it was confirmed that less-immersive VR is more effective in acquiring knowledge in this study, it is expected to maximize the advantages of VR educational programs while overcoming the disadvantages through the design and application of less-immersive, fully-immersive, or hybrid (blended) methods, depending on the purpose of education (whether it is knowledge-oriented or skill-oriented, etc.). 

In this study, self-efficacy was enhanced, but the increase was not statistically significant. First, self-efficacy is an important variable that affects problem-solving motivation and problem-solving ability in learning activities and has a positive effect on confidence in performance [32,33]. In addition, a meta-analytical study conducted on 100 nursing students found that emergency simulation education was effective in enhancing self-efficacy [22]. The interventions examined in this study varied with respect to the number of interventions (i.e., from 1 to 25) and time of intervention (i.e., from 10 min to 45 min). This inconsistency should be addressed in future studies, and the effect of self-efficacy through VR medical education should be re-verified through repeated follow-up RCT studies, to suggest the appropriate time for, and the number of, VR educational interventions. 

The meta-analysis of satisfaction revealed that VR education had a very significant effect on improving satisfaction, with no heterogeneity. However, the study suffered from a limitation with respect to the interpretation of the meaning of the effect size due to the small number of papers that were included in the study. VR education is thought to be more satisfactory than existing learning methods because an immersive experience allows learners to experience reality through multiple senses [34], especially in the medical field, which is almost inaccessible in reality [35]. VR also helps students move away from the traditional education system, which is limited in space and encourages them to work on their own in a different environment. Thus, VR needs to be actively applied in medical education as it can substitute and supplement clinical practice. 

The findings of this study reveal that anxiety was reduced among subjects, but the difference was not statistically significant. Anxiety was first used as a variable in meta-analyses concerning VR education. The anxiety of a learner may lead to lower learning and clinical performance [36]. In a systematic literature review of studies involving 235 nursing students, it was reported that the anxiety level of students who were offered VR education decreased [35]. However, the number of papers included in this study was small, heterogeneity was high, and the intervention time was not consistent (i.e., it varied between 10 min and 150 min). Hence, it is necessary to re-verify the effect on anxiety through repeated follow-up RCT studies.

Our study has another key limitation: In our meta-analysis, there were no studies that presented cost as an outcome value, and we did not identify any previous studies that had systematically reviewed cost at the time of the search [37,38]. Because VR is a new technology and is still evolving, the costs involved may not be reflected in the latest research. Therefore, a systematic review and meta-analysis on cost-effectiveness will be required in the future.

There are currently no standardized evaluation tools, scenarios, or programs for the use of virtual reality in medical education. Therefore, the development of such standardized evaluation methods and scenarios is needed [23]. Furthermore, if a systematic and efficient VR medical education program is developed with due consideration to the cybersickness issue faced by many learners during the early design process, it will greatly assist learners in improving their core competencies. Additionally, if high-quality intervention studies that consider the equipment and computers used in VR education, as well as the instructors’ feedback, are conducted in the future, they may present adequate evidence regarding VR’s effectiveness. Therefore, based on these reviews, it is necessary to develop medical education programs, scenarios, and tools using virtual reality in the future.

## 5. Conclusions

This meta-analysis comprehensively evaluates the use of VR in medical education. As we have reached the post-COVID-19 (endemic) era, medical education is confronting new opportunities, as well as new challenges. Medical education using VR, a digital simulation education method that transcends time and space, can be used to replace or supplement clinical practice education. To support this, we examined RCT studies conducted on current and prospective health personnel and found that VR-applied medical education was effective in improving skill and satisfaction levels. The subgroup analysis revealed that less-immersive virtual intervention was more effective in improving knowledge than fully immersive VR. This review also identified the need for high-quality interventional studies on the problem of cybersickness for VR training and the effective enhancement of learner-acquired skills. By providing medical education that maximizes the advantages of virtual reality based on these findings, learners are expected to have more learning opportunities to complement their limited clinical experience, contributing to the improvement of medical services. 

## Figures and Tables

**Table 1:** Characteristics of selected 25 studies included in the systematic review.

Author (Year) /Country	Study Design	Sample Size			Participants	Age [Mean/Median] (SD/Range)	VR Device (FI/LI)	Feedback	Intervention	Comparator	Session (Total Time)	Outcome	Measurement Tool (Items)
		Total	Exp.	Cont.									
Andreatta et al. (2010)/ USA	RCT	15	7	8	postgraduate year 1 to 4 emergency medicine residents	NR	VR CAVE (FI)	NR	VR triage training	SP (standardized patient)	1 (NR)	Knowledge	Self-developed MCQ (24)
Brinkmann et al. (2017)/ Germany	RCT	36	18	18	medical students	E 23 (21–29) C 23.5 (22–40)	Lap Mentor; Simbionix (LI)	Intensive feedback and individual coaching	VR laparoscopic cholecystectomy	FLS Program	8 (NR)	Skill Self-efficacy	GOALS (5) self-assessment (6)
Cannon et al. (2014)/ USA	RCT	48	27	21	postgraduate year 3 orthopedic residents	NR	ArthroSim (LI)	Tactile feedback	VR arthroscopic knee surgery	standard education	4 (11 h)	Skill	GRS (7) Procedural checklist (21)
Chou et al. (2006)/ USA	RCT	16	8	8	first-year medical students	E 23.9 (2.2) C 23.4 (1.7)	UROMentor; Simbionix (LI)	Tactile feedback	VR ureteroscopy training	TMU (mannequin)	1 (2 h)	Skill	OSATS (6)
Farra et al. (2013)/ USA	RCT	47	22	25	2nd-year associate degree nursing students	(18–57)	3-D Second Life platform (FI)	Provided to the learner note cards and message	VRS in disaster training	web-based modules	1 (20 min)	Knowledge	Validated MCQ (20)
Feifer et al. (2011)/USA	RCT	20	E1:5 E2:5	C1:5 C2:5	medical students	(20–46)	LapSIM; daVinci robotic platform (LI)	NR	VR for robotic surgical training	C1:ProMIS C2:no training	5 (NR)	Skill	MISTELS
Halloran et al. (2017)/ USA	RCT	137	70	67	undergraduate nursing students	27.75 (6.43)	ATI Real Life modules (LI)	NR	Virtual patient simulation for pediatric	no training	2 (2.5 h)	Anxiety	STAI (40)
Jiang et al. (2018)/ China	RCT	46	23	23	3-years anesthesia residents	E 25.1 (1.8) C 25.0 (1.6)	GI-Bronch Mentor; Simbionix (LI)	Tactile feedback and oral instructions	VR fiberoptic bronchoscope manipulation	high-fidelity mannequin	25 (NR)	Skill Confidence	GRS (6) 5-point Likert rating scale
Jung et al. (2012)/ Korea	RCT	114	E1:38 E2:38	38	1-years nursing students	E1 21.13 (3.53) E2 19.08 (0.71) C 19.26 (1.22)	ARSim IV-100 (LI)	Haptic feedback	VR intravenous practical exercise	catheterization on mannequins	1 (10 min)	Anxiety Procedure score	VAS Checklist score (10)
Ko et al. (2018)/ China	RCT	36	12	C1:12 C2:12	junior residents, interns, and elective students	NR	LAP Mentor; Simbionix (LI)	Unclear	VRS for gynecology laparoscopic suturing training	C1:box training C2:no training	2 (4 h)	Skill	GOALS (4)
Kowalewski et al. (2018)/ Germany	RCT	64	33	31	postgraduate year 3–6 senior residents	E 28.1 (2.4) C 28.5 (2.6)	Lap Mentor II; Simbionix (LI)	NR	Multi-modality training (VR with box training) for laparoscopic cholecystectomy	no training	8 (12 h)	Skill	GOALS (5)
Leblanc et al. (2013)/ Canada	RCT	22	11	11	orthopedic surgery residents	(25–40)	Tabs with the haptic device; PHANTOM (LI)	Haptic feedback	VR surgical fixation for ulnar fracture	Sawbones simulator	NR	Skill	GRS (6)
Lucas et al. (2008)/ USA	RCT	32	16	16	first- and second-year medical students	NR	Lap Mentor; Simbionix (LI)	NR	VRT for laparoscopic cholecystectomy	no training	6 (3 h)	Skill Knowledge	OSATS (8)
Nickel et al. (2015)/ Germany	RCT	84	42	42	medical students	E 24.5 (2.6) C 24.1 (2.1)	Lap Mentor; Simbionix (LI)	NR	VRT for laparoscopic cholecystectomy	Blended Learning (10 h of box training + 2 h of E-learning)	3 (12 h)	Skill Knowledge	OSATS (7)
Padilha et al. (2019)/ Portugal	RCT	42	21	21	second-year nursing students	E 19.29 (0.46) C 20.29 (2.19)	VSIM (LI)	NR	Clinical virtual simulation scenario using case-based learning	low-fidelity simulator	1 (45 min)	Knowledge Self-efficacy Satisfaction	True/false and multiple-choice test General self-efficacy scale (10) learner satisfaction with simulation tool (20)
Park et al. (2007)/ Canada	RCT	24	12	12	general surgery and internal medicine residents	NR	AccuTouch colonoscopy simulator (LI)	Haptic feedback	VRT for colonoscopy	no further training	1 (2–3 h)	Skill	GRS (7)
Rossler et al. (2018)/ USA	RCT	20	5	15	prelicensure baccalaureate nursing students	NR	Virtual Electrosurgery Skill Trainer (LI)	NR	VRS for OR fire drill scenario	traditional programmatic education	NR	Knowledge	Self-developed MCQ (7) + true/false test (3)
Stepan et al. (2017)/ USA	RCT	66	33	33	first- and second-year medical students	NR	Oculus Rift VR system, LLC, a VR head-mounted display (FI)	NR	VRS for neuroanatomy	online textbook	1 (20 min)	Knowledge Self-efficacy Satisfaction	Self-developed MCQ (10/30) + fill-in-the-blank questions IMMS (36)
Suebnukarn et al. (2011)/ Thailand	RCT	32	16	16	fourth-year dental students	NR	2.8-GHz Pentium 4PC, with 256 MB RAM and a 13-in computer monitor, connected to two haptic devices (LI)	Haptic feedback	VR for cavity preparation training	training using extracted teeth and phantom head	3 (2 h)	Skill	4-point scale (4)
Tubelo et al. (2016)/ Brazil	RCT	18	9	9	undergraduate dentistry students	NR	Articulate Storyline 2 (LI)	Positive or negative message feedback	Virtual simulation for dentistry	traditional method of education (book)	1 (20 min)	Knowledge	True/false question (10)
Vaccaro et al. (2013)/ USA	RCT	18	9	9	postgraduate years 2–5 resident physicians	NR	DaVinci surgical simulator (FI)	NR	Robotic VRS for surgical skill	Standard Robotic Orientation	1 (3 h)	Skill	GRS (5)
Vargas et al. (2016)/ USA	RCT	36	19	19	medical students	24.9	daVinci Surgical Simulator (FI)	Expert surgeon provided instruction to all participants	VRT for gynecologic surgery of a cystotomy closure	standard education	10 (NR)	Skill	GEARS (5)
Waterman et al. (2016)/ USA	RCT	22	12	10	orthopedic surgery trainees	E (32) C (33)	Arthro VR shoulder simulator; Smbionix (LI)	Haptic feedback	VRS for diagnostic shoulder arthroscopy	standard practice	4 (1 h)	Skill	ASSET (8)
Wilhelm et al. (2002)/	RCT	21	11	10	medical students	E 23.7 (1.8) C 24.2 (1.4)	UROMentor; Simbionix (LI)	Expert endoscopists provided supervised training to the training group	VR case scenario for basic endoscopic performance	no further training	5 (2 h 30)	Skill Self-efficacy	GRS (5) 5-point self-evaluation (10)
Wong et al. (2019)/	RCT	31	16	16	medical students, anesthesia assistants, anesthesia residents	E 54.8 (10) C 55.1 (18)	ORSIM airway simulator (LI)	NR	VR bronchoscopy simulator training to FOB	no further training	1 (1 h)	Skill	GRS (8)

E = Experimental group; C = Control group; VR = Virtual reality; VRS = Virtual reality simulation; VRT = Virtual reality training; FI = Fully immersive virtual reality; LI = Less immersive virtual reality; NR = Not reported; RCT = Randomized Controlled Trials; MCQ = Multiple choice questions; FLS = the Fundamentals of Laparoscopic Surgery; GOALS = the Global Operative Assessment of Laparoscopic Skills score; GRS = global rating scale; TMU = the ureteroscopy training model; OSATS = Objective Structured Assessment of Technical Skills; STAI = the State-Trait Anxiety Inventory; VAS = Visual Analog Scale; IMMS = Instructional Materials Motivational Survey; GEARS = Global Evaluative Assessment of Robotic Skills; ASSET = the Arthroscopic Surgery Skill Evaluation Tool; FOB = Flexible optical bronchoscopic.

## Data Availability

The data presented in this study are available on request from the corresponding author.

## Supplementary Materials

The following supporting information can be downloaded at: https://www.mdpi.com/article/10.3390/ijerph20053895/s1.
