# Supplementary material for: Effects of Medical Education Program Using Virtual Reality: A Systematic Review and Meta-Analysis
Source: Int J Environ Res Public Health. 2023 Feb 22;20(5):3895. doi: 10.3390/ijerph20053895 (PMC10001289; doi:10.3390/ijerph20053895)
Supplement: Supplementary file 1 [file ijerph-20-03895-s001.zip › ijerph-2211137-supplementary.pdf]

Embase

P emtree :

Nurse/mj or physician/mj or nursing student/mj or medical student/mj

P entry :

health professions trainees; Health Occupations Students;

medical student*; nursing student*; undergraduate; prelicensure;

doctor; nurse;

I emtree :

((("Virtual Reality"[Mesh]) OR "Augmented Reality"[Mesh])

I entry :

Virtual Reality; Augmented reality; Mixed reality; Virtual reality simulation training; Virtual simulat*; Virtual education;

[20-04-17]

- Embase

#1. 'nurse'/mj OR 'physician'/mj OR 'nursing student'/mj OR 'medical student'/mj 142477

#2. ‘health professions trainees’:ti OR ‘Health Occupations Students’:ti OR ‘medical student*’:ti OR ‘nursing student*’:ti OR ‘undergraduate':ti OR ’prelicensure’:ti OR ‘doctor’:ti OR ‘nurse’:ti 117456

#3. #1 OR #2 225727

#4. 'virtual reality'/exp OR 'augmented reality'/exp 16771

#5. ‘Virtual Reality’:ti OR ‘Augmented reality’:ti OR ‘Mixed reality’:ti OR ‘Virtual reality simulation

training’:ti OR ‘Virtual simulat*’:ti OR ‘Virtual education’:ti 7552

#6. #4 OR #5 19391

#7. #3 AND #6 **167**

#8. #3 AND #8 AND ([controlled clinical trial]/lim OR [randomized controlled trial]/lim) **16**

CINAHL heading

P :

Nurses; Physicians+; Health Personnel, Unlicensed; Medical Staff; Expert Clinicians+; Students, Medical; Students, Nursing; Students, Dental; Students, Pre-Nursing; Interns and Residents;

P entry :

health professions trainees; Health Occupations Students;

medical student*; nursing student*; undergraduate; prelicensure;

doctor; nurse;

I :

((("Virtual Reality"[Mesh]) OR "Augmented Reality"[Mesh])

I entry :

Virtual Reality; Augmented reality; Mixed reality; Virtual reality simulation training; Virtual simulat*; Virtual education;

[20-04-17]

- CINAHL

#1. (MM "Nurses") OR (MH "Physicians+") OR (MM "Health Personnel, Unlicensed") OR (MM "Medical Staff") OR (MH "Expert Clinicians+") OR (MM "Students, Medical") OR (MM "Students, Nursing") OR (MM "Students, Dental") OR (MM "Students, Pre-Nursing") OR (MM "Interns and Residents") 197,683

#2. TI“health professions trainees” OR TI“Health Occupations Students” OR TI“medical student*”

OR TI“nursing student*” OR TI“undergraduate” OR TI“prelicensure” OR TI“doctor” OR TI“nurse” 109972

#3. #1 OR #2 286336

#4. (MM "Virtual Reality") OR (MM "Augmented Reality") 3690

#5. TI“Virtual Reality” OR TI“Augmented reality” OR TI“Mixed reality” OR TI“Virtual reality simulation training” OR TI“Virtual simulat*” OR TI“Virtual education” 3068

#6. #4 OR #5 5185

#7. "controlled trial" OR "clinical trial" OR "controlled trials“ OR "clinical trials“ OR "randomized" OR "random" OR "trial" OR "trials"

#8. #3 AND #6 **307 / 47**

Cochrane Library

P MeSH :

((((("Students, Nursing"[Mesh]) OR "Students, Medical"[Mesh]) OR "Students, Dental"[Mesh]) OR "Nurses"[Mesh]) OR "Physicians"[Mesh]) OR "Internship and Residency"[Mesh]

P entry :

health professions trainees; Health Occupations Students;

medical student*; nursing student*; undergraduate; prelicensure;

doctor; nurse;

I MeSH :

((("Virtual Reality"[Mesh]) OR "Augmented Reality"[Mesh])

I entry :

Virtual Reality; Augmented reality; Mixed reality; Virtual reality simulation training; Virtual simulat*; Virtual education;

[20-04-17]

- Cochrane

#1. MeSH descriptor: [Nurses] this term only 509

#2. MeSH descriptor: [Physicians] 1 tree(s) exploded 1934

#3. MeSH descriptor: [Internship and Residency] this term only 1203

#4. MeSH descriptor: [Students, Nursing] this term only 389

#5. MeSH descriptor: [Students, Medical] this term only 974

#6. MeSH descriptor: [Students, Dental] this term only 134

#7. #1 or #2 or #3 o #4 or #5 or #6 3398

#8. “health professions trainees”:ti or “Health Occupations Students”:ti or “medical student*”:ti or “nursing student*”:ti or “undergraduate”:ti or “prelicensure”:ti or “doctor”:ti or “nurse”:ti 3387

#9. #7 or #8 6502

#10. MeSH descriptor: [Virtual Reality] explode all trees 144

#11. MeSH descriptor: [Augmented Reality] explode all trees 1

#12. “Virtual Reality”:ti or “Augmented reality”:ti or “Mixed reality”:ti or “Virtual reality simulation training”:ti or “Virtual simulat*”:ti or “Virtual education”:ti 1893

#13. #10 or #11 or #12 1942

#14. #9 and #13 **52 / 50**

Pubmed

#1. ((((("Nurses"[Majr]) OR "Physicians"[Majr]) OR "Internship and Residency"[Majr]) OR "Students, Nursing"[Mesh]) OR "Students, Medical"[Mesh]) OR "Students, Dental"[Mesh] 252123

#2. (((((((health professions trainees[Title/Abstract]) OR Health Occupations Students[Title/Abstract]) OR medical student*[Title/Abstract]) OR nursing student*[Title/Abstract]) OR undergraduate[Title/Abstract]) OR prelicensure[Title/Abstract]) OR doctor[Title/Abstract]) OR nurse[Title/Abstract] 236019

#3. #1 or #2 424985

#4. ("Virtual Reality"[Mesh]) OR "Augmented Reality"[Mesh] 1743

#5. (((((Virtual Reality[Title/Abstract]) OR Augmented reality[Title/Abstract]) OR Mixed reality[Title/Abstract]) OR Virtual reality simulation training[Title/Abstract]) OR Virtual simulat*[Title/Abstract]) OR Virtual education[Title/Abstract] 12019

#6. #4 OR #5 12405

#7. #3 and #6 **960 -> 186**
